# Early maladaptive schemas from child maltreatment in depression and psychotherapeutic remediation: a predictive coding framework

**DOI:** 10.3389/fpsyt.2025.1548601

**Published:** 2025-04-29

**Authors:** Gita Ramamurthy, Alan Chen

**Affiliations:** ^1^ Department of Psychiatry, Alan and Marlene Norton College of Medicine, SUNY Upstate Medical University, Syracuse, NY, United States; ^2^ Keck School of Medicine, University of Southern California, Los Angeles, CA, United States

**Keywords:** early maladaptive schemas (EMS), depression, predictive coding, child maltreatment (CM), psychotherapy

## Abstract

Schemas are affective-cognitive conceptual models of self, others and the world, derived from life experience. Predictive Coding theory proposes schema are created from perceptual input as follows: Based on previous similar experiences, the brain generates schema, with “predictions,” expectations of future sensory experiences. Discrepancy between predicted versus actual experience produces a “prediction error.” Exposure to prediction errors considered more certain than the predictions of a schema prompts the hippocampus to update and revise the schema. Hypothesized underlying mechanisms include memory reconsolidation, extinction and pattern separation. Depression is characterized by negative schemas predicting helplessness, hopelessness and worthlessness. Early maladaptive schemas, from childhood, are implicated in mediating the greater risk of depression from childhood maltreatment. Prominent examples include the Defectiveness/Shame self-schema, predicting a flawed, unlovable self and the Social Isolation/Alienation schema, predicting isolation. Predictive Coding offers the following biopsychosocial hypothesis explaining how childhood maltreatment promotes depressogenic early maladaptive schema, and how psychotherapy can help: Schema can be difficult to change because of an attention/memory bias away from schema-incongruent information that generate prediction errors prompting schema revision. Childhood maltreatment exacerbates this learning bias. Maladaptive coping styles associated with childhood maltreatment, decrease exposure to experiences contradicting depressogenic schema. Biological changes from childhood maltreatment, including inflammation, interfere with hippocampal updating of schema. Finally, impaired socio-occupational function, associated with childhood maltreatment, reinforces depressogenic schema. By targeting factors associated with childhood maltreatment, which reinforce depressogenic early maladaptive schema or diminish prediction errors, psychotherapy can facilitate revision of depressogenic schema.

## Child maltreatment, early maladaptive schemas, and predictions

A history of child maltreatment (CM), involving abuse (emotional, physical, or sexual) or neglect, increases the risk for depression incidence, severity, and treatment resistance (1–5). Predictive coding, a prominent neuroscience hypothesis, may explain how early maladaptive schemas (EMS) from CM promote depression and are addressed by psychotherapy.

Schemas are mental models, characterizing broad, core affective and cognitive beliefs about self, others, and the world (6). By organizing and filtering information for attention, interpretation, and memory, schemas can facilitate learning and decision making (7–9). Importantly, schemas generate *predictions* of cognitive, affective, and perceptual states typically characterizing different contexts (6). Thus, predictions from schemas offer guidance for future navigation of such contexts.

Childhood schemas can have long-standing psychological consequences (10). Bowlby proposed that “attachment styles,” reflecting child–parent relationships, substantially influence adult schemas of self (self-schemas), and of others (10). Emotionally attuned parenting facilitates the development of a “secure” attachment style (11). Securely attached adults generally “predict” being a lovable, competent self, and trustworthy and accepting close others (11). Such positive schemas of self/others are understood to foster social support and psychological health (11–13). In contrast, CM promotes EMS of self/others (14), and insecure attachment (15), which substantially contributes to psychopathology (15–17).

### Depression, early maladaptive schema, and interlocking self-reinforcing feedback loops

Depression is characterized by negative schemas, predicting helplessness, hopelessness, and worthlessness (18, 19). Depressogenic EMS are implicated in increased depression risk from CM (20). In a recent metanalysis, the Defectiveness/Shame and Social Isolation/Alienation EMS were most strongly associated with depression. The Defectiveness/Shame self-schema predicts an inferior, unlovable self. The Social Isolation/Alienation self-schema predicts not belonging (21). Shame appears particularly important in perpetuating depression, through maladaptive styles of coping with shame, including avoidance (flight), overcompensation (fight), and surrender (freeze) (21, 22).

Surrendering to the Defectiveness/Shame EMS involves accepting shame, harsh self-criticism, and predicting failure and social rejection (22). Shame-prone persons often conceal personal “defectiveness,” even from themselves (23, 24). Overcompensation coping involves hostile, grandiose defensive denials of shame. Avoidance coping minimizes consciousness of shame by diverting attention, through compulsive reward-seeking (addiction), self-injury, and experiential avoidance (suppression) of distress (25).

Securely attached persons characteristically acknowledge and process distress with self-compassion and by seeking social support (11–13). In contrast, CM is associated with harsh self-criticism (26), the Defectiveness/Shame EMS (14), avoidant coping (27) and insecure attachment (15), which all promote depression (20, 28–32).

Impaired occupational function (33) and loneliness (34, 35) associated with CM may be mediated by insecure attachment, shame, and maladaptive shame-coping strategies (36–41). Interpersonal difficulties appear to mediate the association of childhood adversities with depression (42–44). Low social connectedness promotes depression risk, severity, and treatment resistance (45–47). Social support (48) is protective.

The Defectiveness/Shame and Social Isolation/Alienation EMS appear to negatively impact interpersonal function among persons with CM (49). The adverse impact is attributed to impaired mentalization, the ability to understand mental states in self/others (50, 51). Avoidance of self-disclosure in shame-prone persons, which promotes social disconnection, may also contribute (52).

**Figure 1:**
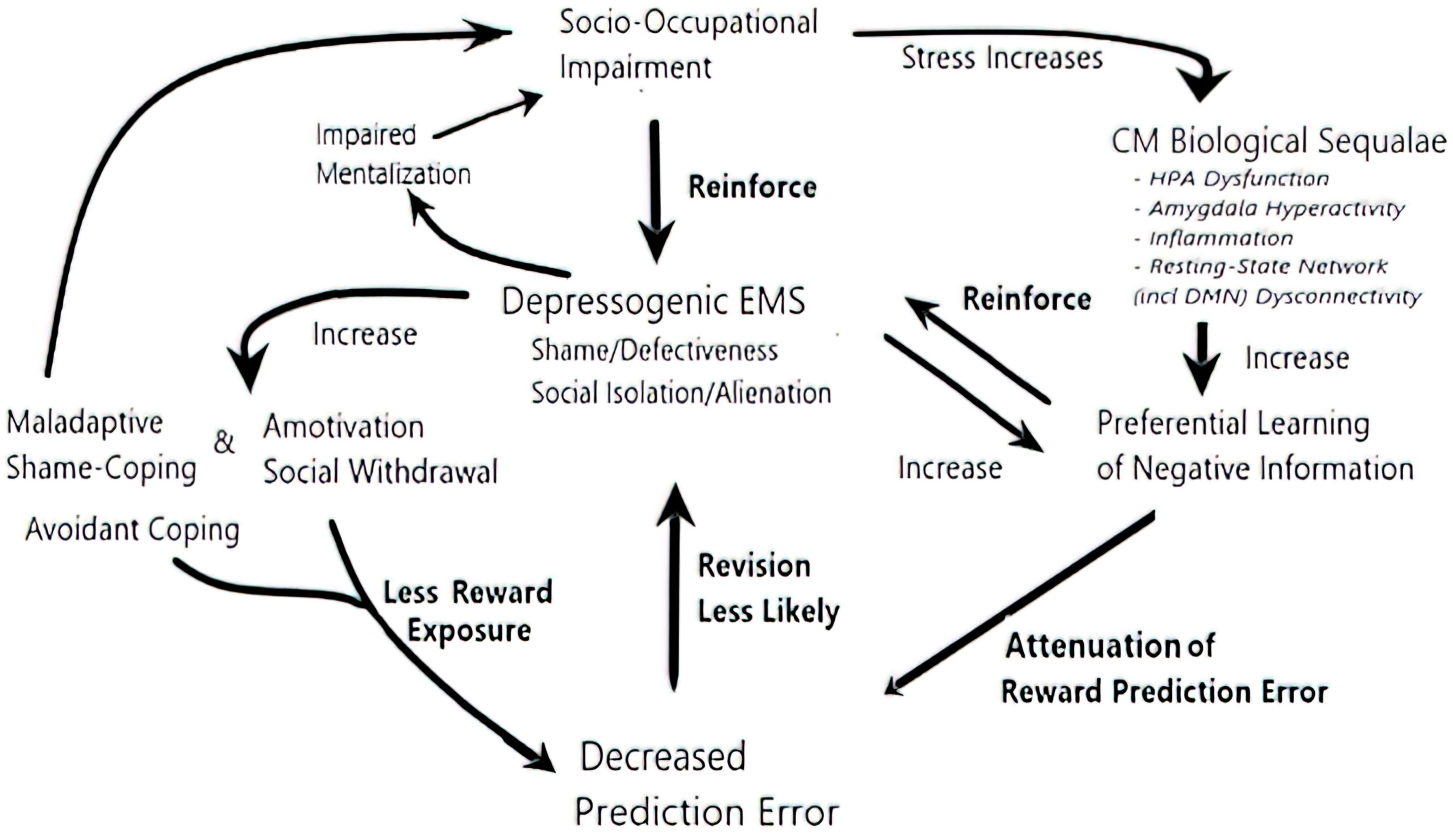
The self-sustaining feedback loops underlying depression. CM exacerbates all the elements driving the loops. In one loop, psychological sequelae of CM, including shame, maladaptive shame-coping, mentalization difficulties, social withdrawal, and a motivation, all impair socio-occupational function (36–41). In turn, repeated socio-occupational disappointments can confirm EMS of Defectiveness/Shame and Social Isolation/Alienation, reinforcing their certainty and resistance to schema-incongruent information (53, 54). In another interlocked recursive loop, CM sequelae diminish exposure to and learning from reward prediction errors, which could have revised depressogenic EMS: For example, avoidant coping decreases social/occupational engagement, thus reducing opportunities for reward prediction errors. When rewarding experiences do arise, their impact is decreased by biological sequelae of CM: amygdala hyperactivity, HPA axis dysfunction, and inflammation promote preferential learning of negative information (55–59), through attenuated reward prediction error, negative overgeneralized memory, and negative memory/attention biases socio-occupational stress further exacerbates the biological sequelae (60–62), completing the loop.

Socio-occupational disappointments would, reasonably, be expected to confirm depressogenic schema (53), including the Shame/Defectiveness and Social Isolation/Alienation EMS. These schemas support self-sustaining feedback loops (**Figure 1**) underlying depression.

## An introduction to predictive coding

Predictive coding offers a biopsychosocial framework, explaining how CM promotes depressogenic EMS and how psychotherapy can help. Principles underlying predictive coding were first characterized in sensory processing, explaining how the brain constructs reality from perception.

Predictive coding theory proposes that the brain creates predictions of reality from schemas and perceptual input as follows (63–67): based on schemas, derived from repeated associative memory patterns (see below), the brain generates “predictions,” expectations of future sensory experiences. Discrepancies between the prediction and actual experience produce “prediction errors,” signaling mismatch. The brain minimizes prediction errors, through “active inference,” a process that optimizes the match between actual and predicted experience. Active inference involves either updating the pre-existing prediction or, instead, suppressing the prediction error.

Predictions and prediction errors are inherently uncertain, derived from statistically “noisy” environmental signals. Predictions cannot be perfectly accurate. The level of certainty in predictions, relative to prediction error, determines if, and how much, the prediction is updated following an experience (68).

When an experience that is incongruent with the prediction is considered more accurate than the prediction, the prediction error is *more certain* than the pre-existing prediction. The prediction, consequently, is updated (68). Prediction errors, from information considered as *less certain* than the prior prediction, are instead suppressed by changing neural input to match the prediction (68, 69). Commonly, attention is redirected away from the perceptions generating the prediction error. By ignoring unexpected sensory information, the prediction error is dismissed. Thus, the pre-existing prediction is maintained rather than being updated (70, 71).

Highly certain predictions are harder to update because, comparatively, prediction errors appear less certain and are, therefore, suppressed. Disregarding prediction errors, reflecting anomalous, “noisy,” incongruent information, is adaptive when the prior prediction is, indeed, more accurate. However, ignoring unexpected perceptions (dismissing “prediction errors”) that better reflect reality than pre-existing predictions is maladaptive.

Predictive coding in perceptual processing is better understood than in processing memory and schemas. Nonetheless, emerging research shows clear parallels in the principles of predictive coding in perception, memory and schemas (71–75). Hypotheses suggest that psychotherapy often promotes “prediction errors” from unexpected experiences, which then revise maladaptive schemas of self/others, derived from autobiographical memories of childhood adversity (71–75).

### Schemas

Autobiographical memories are believed to be represented by neural networks, with distributed nodes across brain regions bound together through Hebbian plasticity (76). Specialized nodes represent different elements of memory, such as the sensations, emotions, and spatiotemporal context. Over time, ventromedial prefrontal cortex (vmPFC)—hippocampus interactions “schematize,” or transform, memories into schemas (77). When schemas generate faulty predictions, the prediction error can prompt hippocampal updates to memories and schema (78–80).

The hippocampus creates memories by rapidly connecting associated experiences (80). Over time, vmPFC-hippocampal interactions are hypothesized to extract patterns of common, recurrent associations, thereby creating conceptual structures of cognitive-affective knowledge or schemas (67, 80). Schematization allows information reflecting memory commonalities to be condensed, organized, and recalled efficiently (67, 77, 81–84). Unique details become less accessible (7). Schemas also generate predictions, since common, repeated associations (“statistical regularities”) generally recur.

For example, seasoned travelers predict the common, repeated aspects of airport travel, involving typical sequences, tasks, emotions, and so forth. Recurrent, common aspects of airport memories become incorporated into airport travel schema, and associated predictions, while excluding unique features, such as flight numbers.

### Cognitive-affective schema after childhood trauma and depression

EMS in the Disconnection/Rejection Domain, such as the Defectiveness/Shame and Social Isolation/Alienation EMS, are proposed to develop from schematization of repeated childhood experiences of unmet core emotional needs from attachment figures (85, 86).

Depressogenic EMS is proposed to arise from extracting common, repeated experiences of CM, woven together with schema of self/others. Children may respond to harsh parenting with habitual submission (86, 87). Repeated appeasement of belittling, dominant others can lead to an internalized shame-based self-schema, consistent with the Defectiveness/Shame EMS (86, 87). Recurrent parental maltreatment is believed to lead to schema of others as rejecting (Social Isolation/Alienation EMS) (11, 86, 88).

### Depressogenic early maladaptive schema—neurodevelopmental hypotheses

CM-induced neurodevelopmental changes to the circuitry are hypothesized to promote vulnerability to EMS (85). Mapping the underlying circuity or changes from CM promoting EMS remains preliminary. Research challenges are attributed to heterogeneous timing and type of CM and genetic susceptibility (89).

CM-induced changes in the structure, function, and connectivity of brain areas in prefrontal-subcortical circuits (90), such as the mPFC, hippocampus, limbic areas, Default-Mode Network (DMN), involved in self-referential processing (91, 92), and mentalizing network (93), are hypothesized to increase vulnerability to maladaptive schema of self/others (85). Amygdala hyper-reactivity (2, 94, 95), altered amygdala connectivity with DMN, and mentalizing network nodes (2, 96) and hippocampal volume reduction (2) are proposed to increase cognition, salience, and recall of negative self-characteristics. Blunted striatal reward activity (97) and increased threat-related insula and amygdala activity (98) associated with CM may contribute to impaired self-efficacy (99, 100). CM-induced changes to mentalizing networks are suggested to play a role in schema predicting untrustworthy others (101).

In summary, CM-induced neurodevelopmental changes are hypothesized to promote negative schema of rejecting/untrustworthy others and self-schema involving negative self-characteristics. These negative self-/other schemas are consistent with the Defectiveness/Shame and Social Isolation/Alienation EMS. Established depressogenic EMS are self-reinforcing: vmPFC-hippocampal interactions boost engagement toward schema-congruent information (7, 67). CM-induced cognitive bias toward negative information provides further confirmation.

### Child maltreatment promotes preferential learning of negative information

#### Overgeneralization, pattern completion, and impaired pattern separation

Sensory, affective, and cognitive aspects of predictions are encoded by diverse specialized neural areas, or nodes, interconnected within an auto-associative network. A partial cue activating several nodes can activate the entire network. For example, packing luggage can instantiate a schema of airport travel, with characteristic sensory, cognitive, and emotional aspects. Instantiating a prediction from a partial cue is termed “Pattern Completion.” (102).

Pattern Completion is counterbalanced by Pattern Separation, a neurocomputational process that encodes new memories distinctly from old memories in prior contexts, thereby minimizing interference (103). When Pattern Separation is impaired, Pattern Completion can become maladaptive, promoting overgeneralization (104). Overgeneralized learning extrapolates associations from isolated experiences to inappropriate, unrelated contexts (105): for example, a depressed patient may receive mild criticism about performance in an atypical circumstance. This partial cue of isolated criticism, in a narrow, atypical context, can instantiate a childhood schema of self-denigration, related to harsh parenting (105). Thus, overgeneralization can reinforce the Defectiveness/Shame EMS.

Negative memory overgeneralization likely mediates the association of CM with pessimism and harsh self-criticism (106). Hippocampal neurogenesis is implicated in Pattern Separation (103, 107, 108). CM-induced inflammation and hypercortisolemia can suppress neurogenesis (103, 109), thereby interfering with Pattern Separation. Such pathophysiological changes are believed to underlie increased overgeneralization in CM (103).

#### Negative attention and memory bias

In addition to their role in schematization, vmPFC—hippocampus interactions are proposed to underlie a top-down attention/memory bias toward schema-congruent information (7, 67): new information, congruent with a schema, is easier to attend and recall than schema-incongruent information (67).

The learning bias against schema-incongruent information may reflect skepticism of anomalous data. Skepticism about data that is inconsistent with statistical regularities embedded in schemas can be adaptive. However, bias against schema-incongruent information is maladaptive when the schema is held with unwarranted certainty.

CM biases attention and memory toward negative (over positive) information. Examples include angry over happy faces or negative (over positive) self-descriptive words (110, 111). Negative attention bias predicts increased depression severity (112). Negative memory bias is associated with rumination, which in turn, exacerbates depression (113). Biological sequelae attributed to CM, including amygdala hyperactivity, resting-state network (including DMN) dysconnectivity (114), HPA axis dysfunction, and increased inflammation (2, 55, 95, 115), are implicated in negative attention and memory bias (56–58, 95, 116, 117).

#### Attenuated reward prediction errors, child maltreatment and preferential learning of negative information—interim summary

Prediction errors from unexpected rewards would be expected to prompt revision of depressogenic schema (78, 79). Growing evidence indicates that CM diminishes reward prediction errors, thereby disrupting learning from positive experiences (97). In support of this connection, attenuated reward prediction errors are linked to anhedonia and a higher depression risk (118).

CM likely promotes preferential learning of negative information through diminished reward prediction errors, overgeneralized memory, and negative learning biases, thereby promoting depression (106, 110, 113).

Preferentially attending to and remembering failure and rejection, while minimizing socio-occupational rewards, promotes a motivation and social withdrawal. Furthermore, these learning disruptions confirm depressogenic EMS, such as Defectiveness/Shame and Social Isolation/Alienation. The mutual reinforcement of depressogenic schema and negative learning biases creates another positive feedback loop in depressed patients with CM (**Figure 1**).

### Child maltreatment and interlocking self-reinforcing feedback loops promote depressive early maladaptive schema

Many forms of psychopathology, including depression, are proposed to result from maladaptive predictions carrying excessive certainty over prediction errors, rendering them resistant to update by new incongruent information (54, 119–121).

CM exacerbates interlocking self-reinforcing feedback loops proposed to maintain depression (53) (**Figure 1**). Repeated life disappointments can confirm EMS of Defectiveness/Shame and Social Isolation/Alienation, increasing their certainty and resistance to schema-incongruent information (53, 54). CM exacerbates this feedback loop, in which socio-occupational dysfunction and excessive certainty in depressogenic EMS, such as the Defectiveness/Shame and Social/Isolation/Alienation EMS, cumulatively reinforce each other.

Both biological and psychological sequelae of CM promote and reinforce depressogenic EMS (2, 122): for example, harsh self-criticism, impaired mentalization, and negative cognitive biases amplify the impact of aversive experiences, while avoidant coping diminishes exposure to, and learning from, prediction errors generated by social/occupational rewards (2, 122). Finally, biological sequelae of CM also amplify learning reinforcing depressogenic EMS, while attenuating reward prediction error (55–62).

## Prediction updating by psychotherapy

### Neuroscience hypotheses of therapeutic change

The relative imperviousness of EMS to schema-incongruent information effectively impairs learning impeding therapeutic change in persons with CM.

Neural processes proposed to mediate prediction updating in psychotherapy include memory reconsolidation, extinction, and pattern separation (123–125). These processes are currently understood as follows: Prediction errors, sent to the hippocampus, appear to prompt updates to neural networks that encode predictions (126, 127). Memory reconsolidation involves erasing existing maladaptive associations and creating new associations (123, 124, 128). Extinction involves creating a new memory that competes with the old maladaptive memory (123). Pattern separation encodes a new adaptive memory in a distinct context, separate from the prior context, to counteract overgeneralization (125, 129).

In summary, extinction and pattern separation lead to coexisting old and new predictions. Memory reconsolidation replaces old predictions with new ones.

### EMS disrupt therapeutic relationships

The therapeutic alliance is the chief predictor of successful psychotherapy adherence (130) and outcomes (131). CM sequelae, including shame/harsh self-criticism (132–134) and insecure attachment (15, 135, 136), can disrupt the therapeutic bond. This disruption likely contributes to treatment resistance in persons with CM (4, 5).

The Defectiveness/Shame EMS may disrupt the therapeutic alliance in several ways. When patients cope with shame through denial, withdrawal, self-criticism, or hostility, therapists may respond by distancing (137). Furthermore, shame induces the urge to avoid self-disclosure (24). Shame-prone persons reveal less in psychotherapy (138, 139).

### The therapeutic alliance and prediction errors

A “corrective emotional experience” is an unexpected experience with a therapist, which challenges maladaptive schemas, and is believed to generate a prediction error (140–143). This “corrective emotional experience” is considered an important mechanism of change in psychotherapy (123, 140, 144). For example, feeling safety, support, and connection with a therapist is incongruent with depressogenic EMS predicting inadequacy and rejection (145). “Attachment security priming,” in which a therapist is experienced as a safe, secure attachment figure (146), contributes substantially to the healing benefit of the therapeutic alliance (145).

Attachment security priming alleviates many factors driving self-reinforcing feedback loops underlying depression. Security priming increases feelings of self-compassion, self-acceptance, and belonging (145). These feelings can generate prediction errors, directly counteracting the EMS of Defectiveness/Shame and Social Isolation/Alienation, of a flawed, inferior, unlovable self (21).

Security priming may improve receptivity to schema-incongruent information, particularly in persons with CM, given their higher prevalence of insecure attachment (147). Insecurely attached persons characteristically ignore or defend against new schema-incongruent information (11), which would be expected to diminish exposure to prediction errors. Security priming has been proposed to promote epistemic trust, the willingness to consider new perspectives from a therapist as trustworthy, generalizable, and self-relevant (148). Security priming also increases cognitive openness to new information (145). Epistemic trust and cognitive openness both likely support receptivity to schema-incongruent information and revision of maladaptive schema.

### Psychotherapy targets elements of self-reinforcing feedback loops

Psychological interventions may improve biological sequelae of CM. Amygdala hyperactivity and inflammation, which are implicated in negative learning bias (56, 57, 59, 117, 149, 150), improve with security priming and cognitive behavioral therapy (CBT), respectively (149–151).

**Figure 2:**
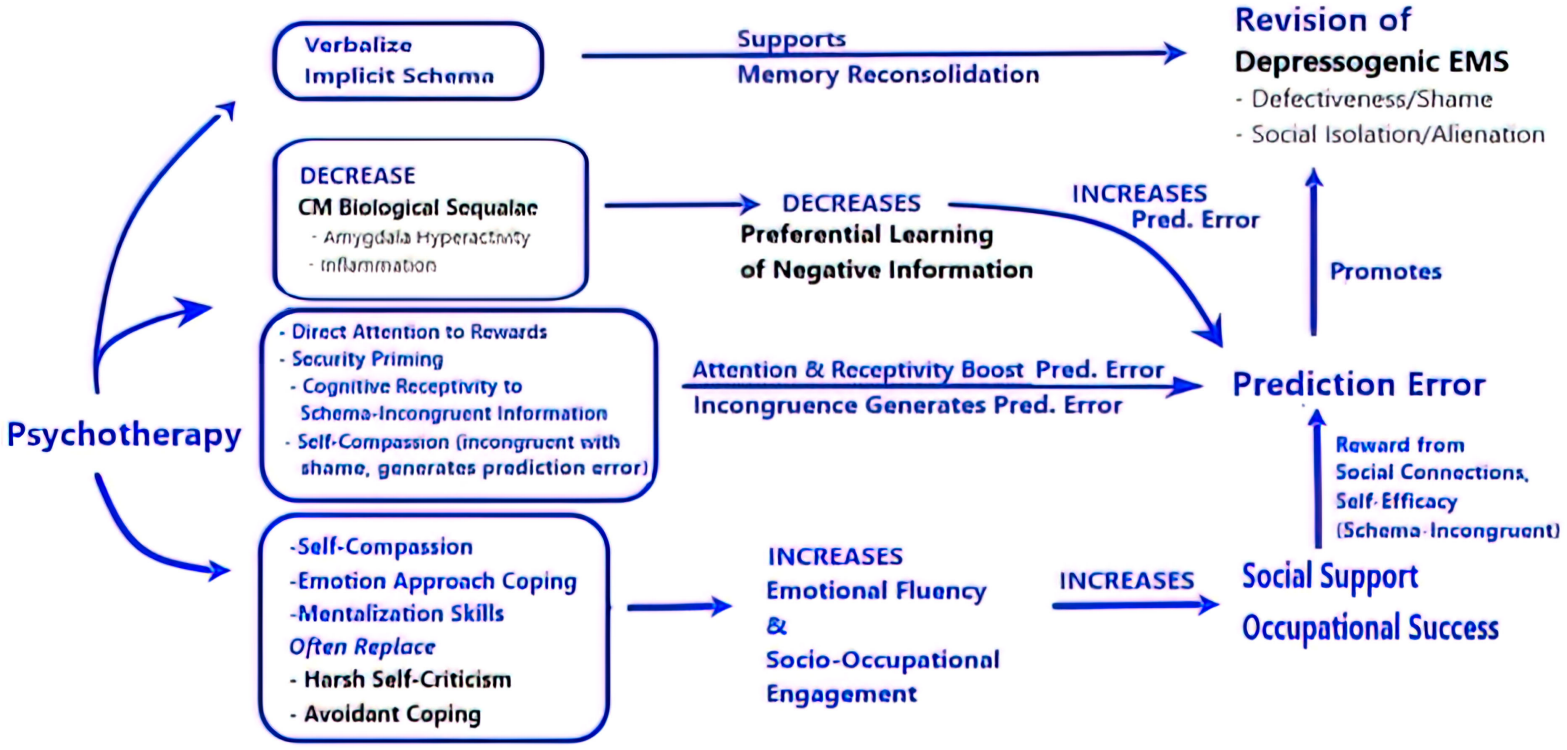
Psychotherapy targets elements promoting EMS and boosts factors supporting revision of depressogenic early maladaptive schema. The figure illustrates hypothetical pathways explaining how psychotherapy targets vulnerability factors (in black) promoting depression/depressogenic and boosts factors (in blue) supporting EMS revision. (1) Verbalizing implicit schema supports memory reconsolidation. (2) Psychotherapy (security priming & CBT) improves CM biological sequelae that would otherwise attenuate prediction error. (3) Prediction error prompting EMS revision is boosted by directing attention and improving cognitive receptivity to schema-incongruent information. Security priming also promotes self-compassion, which is incongruent with the Defectiveness/Shame EMS, and thus generates a prediction error. (4) Self-compassion, emotion approach coping, and Mentalization skills counteract harsh self-criticism and avoidant coping, support emotional fluency, and facilitate socio-occupational engagement.

Different psychotherapy approaches target different elements of the self-reinforcing feedback loop (**Figure 2**). Interpersonal therapy facilitates social engagement over withdrawal (152). CBT challenges preferential learning of negative information and anhedonia/social withdrawal by encouraging engagement with rewarding experiences (153). Attitudes of acceptance, mindfulness, self-compassion, and committed action in Acceptance and Commitment Therapy counteract avoidant coping and the Defectiveness/Shame EMS (154, 155).

### Psychotherapy revises EMS

Several psychotherapy schools, including CBT, schema therapy and many psychodynamic approaches directly target maladaptive schemas for revision and updating. One first step involves helping patients verbalize implicit maladaptive schemas. This may support memory reconsolidation (128): memory reconsolidation does not impact inactive predictions stored in memory but rather affects actively recalled predictions. Explicitly articulating EMS may support active recall, thereby facilitating reconsolidation.

Prediction error appears important for hippocampal prediction updating (156), and thus, important for revising EMS. Indeed, pretreatment reward prediction errors predict depression responsiveness to CBT (157). Exposure to prediction errors appears diminished by both avoidant coping and preferential learning of negative information, which are targets of psychotherapy (153, 154). Attention to schema-incongruent information, by CBT and psychodynamic therapies (74, 158–160), may instead boost the neural encoding of prediction errors (161).

### Summary: experiential avoidance, impaired socio-occupational function and depressogenic EMS are mutually reinforcing, and key targets of psychotherapy

The Defectiveness/Shame EMS is hypothesized to reflect the maladaptive persistence of an appeasement strategy toward harsh parents, accompanied by experiential avoidance of anger and grief (87). Experiential avoidance can be understood as a maladaptive prediction, that anger and grief are so shameful, dangerous, and/or intolerable that suppression is the only solution (162). Many psychotherapy approaches encourage “emotion approach coping,” attending to and processing emotions, instead of suppression (153, 154, 163, 164).

In psychotherapy, patients may discover they can tolerate their anger and grief. This prediction error would be expected to revise habitual experiential avoidance to a new prediction: attention to affective information is meaningful and useful.

Access to a wider emotional range can expand a patient’s repertoire to include acknowledging and communicating distress, improved mentalization, assertiveness, and social engagement. Greater self-compassion and emotional fluency enable social and occupational success, which decreases amygdala activity (165) and inflammation (166) and leads to further positive experiences (reward prediction errors). Psychotherapy can thus facilitate a virtuous self-reinforcing cycle of more positive self-schema, adaptive coping strategies and improving function.
